# Presence of both alterations in FGFR/FGF and PI3K/AKT/mTOR confer improved outcomes for patients with metastatic breast cancer treated with PI3K/AKT/mTOR inhibitors

**DOI:** 10.18632/oncoscience.307

**Published:** 2016-06-30

**Authors:** Jennifer J. Wheler, Johnique T. Atkins, Filip Janku, Stacy L. Moulder, Philip J. Stephens, Roman Yelensky, Vicente Valero, Vincent Miller, Razelle Kurzrock, Funda Meric-Bernstam

**Affiliations:** ^1^ Translational clinical oncology, Novartis Pharmaceuticals, Cambridge, MA, USA; ^2^ Department of Investigational Cancer Therapeutics, The University of Texas MD Anderson Cancer Center, Houston, TX, USA; ^3^ Department of Breast Medical Oncology, The University of Texas MD Anderson Cancer Center, Houston, TX, USA; ^4^ Foundation Medicine, Cambridge, MA, USA; ^5^ The Center for Personalized Cancer Therapy, University of California San Diego Moores Cancer Center, La Jolla, CA, USA

**Keywords:** breast cancer, FGFR, next-generation sequencing, PI3K

## Abstract

There is limited data on co-expression of FGFR/FGR amplifications and PI3K/ AKT/mTOR alterations in breast cancer. Tumors from patients with metastatic breast cancer referred to our Phase I Program were analyzed by next generation sequencing (NGS). Genomic libraries were selected for all exons of 236 (or 182) cancer-related genes sequenced to average depth of >500× in a CLIA laboratory (Foundation Medicine, Cambridge, MA, USA) and analyzed for all classes of genomic alterations. We report genomic profiles of 112 patients with metastatic breast cancer, median age 55 years (range, 27-78). Twenty-four patients (21%) had at least one amplified FGFR or FGF. Fifteen of the 24 patients (63%) also had an alteration in the PI3K/ AKT/mTOR pathway. There was no association between alterations in FGFR/FGF and PI3K/AKT/mTOR (*P*=0.49). Patients with simultaneous amplification in FGFR/FGF signaling and the PI3K/AKT/mTOR pathway had a higher rate of SD≥6 months/PR/ CR when treated with therapies targeting the PI3K/AKT/mTOR pathway than patients with only alterations in the PI3K/AKT/mTOR pathway (73% vs. 34%; *P*=0.0376) and remained on treatment longer (6.8 vs. 3.7 months; *P*=0.053). Higher response rates were seen in patients with simultaneous amplification in FGFR/FGF signaling and alterations in the PI3K/AKT/mTOR pathway who were treated with inhibitors of that pathway.

## INTRODUCTION

Fibroblast growth factor receptors (FGFR) and their ligands (FGF) play a critical role in proliferation, migration, angiogenesis, and survival of cancer cells. FGFR signaling has been primarily described as an escape mechanism of vascular endothelial growth factor (VEGF) targeted therapies, but recent studies have also identified alterations in FGFRs as driving oncogenes in certain tumor types [1, 2]. Alterations in FGFR signaling include, but are not limited to, gene amplification or post-transcriptional regulation leading to receptor overexpression; FGFR mutations producing receptors that are either constitutively active or have a reduced dependence on ligand binding for activation; and upregulation of FGF expression and the enhanced release of FGFs from the extracellular matrix, resulting in paracrine/autocrine activation of the pathway [3]. Dysregulation of FGFR signaling can lead to downstream activation of mitogen activated protein kinase (MAPK) and phosphoinositide-3-kinase (PI3K)/AKT pathways [2, 4, 5]. FGFR signaling also interacts with phospholipase C-gamma (PLC-γ) to stimulate protein kinase C (PKC), which phosphorylates RAF in the MAPK pathway [2].

There are four identified FGFR receptors, FGFR1, FGFR2, FGFR3, and FGFR4 that bind to a variety of FGFs. Twenty-two different FGFs have also been identified. *FGF1* through *FGF10* all bind to FGFRs. *FGF11* through *FGF14* have similar homologies to the other FGFs, but do not bind to FGFRs and have distinct functional differences. *FGF19*, *FGF21*, and *FGF23* are considered hormone-like and, in contrast to the local activity of the other FGFs, have systemic effects. The different FGFs and their corresponding receptors are expressed in a tissue specific manner, contributing to the specificity of the ligand-receptor interaction [2, 6].

Members of the FGFR family are rarely mutated but frequently amplified or overexpressed in breast cancer, which is often accompanied by increase, or altered, expression of FGF ligands [7]. Hybrid capture based broad next-generation sequencing (NGS) has allowed us to take an in-depth look at the genomic landscape of breast cancer patients seen in our phase I clinic [8]. The purpose of this study was to estimate the frequency of alterations in FGFRs and FGFs and to characterize the nature of these alterations in a population of patients with advanced, heavily pretreated breast cancer. A secondary objective was to report on any associations between molecular profile and response to targeted therapy.

## RESULTS

### Patients

A total of 112 patients with advanced breast cancer had their tumors analyzed by Foundation Medicine either prospectively to determine an appropriate clinical trial with targeted therapy or retrospectively to correlate with response to therapy. Median age was 55 years (range, 27 to 78 years). Ninety patients (80%) were white; nine (8%) were African American; ten (9%) were Hispanic; and, four (3%) were Asian. Fifty-five patients (49%) were hormone receptor (HR)-positive (estrogen or progesterone) and eight (7%) were HER2-positive. Detailed patient characteristics are listed in Table 1.

**Table 1:** Histopathologic and Molecular Characteristics of 24 Patients with Amplifications in FGFR/FGF signaling

Case No.	Histology	ER status	PR status	HER2 status	Biopsy Location	Molecular Profile
1	Ductal	Positive	Positive	Negative	Liver	ATM mutation R189K, AURKA amplification, CCND1 amplification, FGF19 amplification, ZNF703 amplification
2	Ductal	Positive	Positive	Negative	Breast	PIK3CA mutation H1047R, CCND1 amplification, MCL1 amplification, EMSY amplification, FGF19 amplification, FGF3 amplification, FGF4 amplification
3	Metaplastic	Negative	Negative	Negative	Breast	PIK3CA amplification, PTEN deletion, FGFR2 amplification, MYC amplification, TP53 mutation R273C
4	Ductal	Negative	Negative	Negative	Chest wall	FGFR2 amplification, TP53 mutation R306*, CDH1 mutation Q264*
5	Ductal	Positive	Positive	Negative	Lymph node	AKT1 mutation E17K, KIT amplification, FGFR1 amplification, MYC amplification, TP53 mutation P36fs*7, NFKBIA amplification, BCL2L2 amplification, ZNF703 amplification
6	Ductal	Positive	Positive	Negative	Metastasis	PIK3CA mutation H1047R, PIK3R1 mutation G376R, CCND1 amplification, FGFR1 amplification, MYC amplification, MCL1 amplification, FBXW7 mutation D112E
7	Ductal	Negative	Negative	Negative	Liver	FGF14 amplification, GATA3 mutation A333fs*20, IRS2 amplification
8	Ductal	Negative	Negative	Negative	Breast	TSC2 mutation G1055fs*113, FGFR1 amplification, ATM mutation V1569fs*29, MCL1 amplification, TP53 mutation L111fs*40, MYC amplification, MYST3 amplification
9	Ductal	Negative	Negative	Negative	Breast	CDKN2A mutation Y44fs*1, TP53 mutation, CCND1 amplification, CCND2 amplification, FGF19 amplification, FGF3 amplification, FGF4 amplification, FGF6 amplification
10	Ductal	Negative	Negative	Negative	Breast	PTEN deletion, FGFR1 amplification, CCNE1 amplification, MCL1 amplification, MYC amplification, TP53 mutation Y234*, BCL2L2 amplification, NPM1 truncation 5′UTR, FAM123B mutation G303D
11	Ductal	Positive	Positive	Negative	Metastasis	CCND1 amplification, FGFR1 amplification, PRKDC rearrangement, PTEN protein loss
12	Ductal	Negative	Negative	Negative	Breast	FGFR1 amplificaiton, FGFR2 amplification, CCND1 amplification, MAP2K2 amplification, MYC amplification, TP53 mutation C242fs*5, PTEN protein loss
13	Ductal	Positive	Positive	Negative	Breast	CCND1 amplification, FGFR1 amplification, ARID1A mutation Q708*
14	Metaplastic	Negative	Negative	Negative	Breast	PIK3R1 mutation Y580fs*19, CCND2 amplification, CDKN2A deletion, FGF23 amplification
15	Ductal	Positive	Negative	Negative	Liver	PIK3CA mutation E454K, BRCA2 mutation K3326*, CCND1 amplification, HRAS mutation G12D, GATA3 mutation D336fs*17, FGF19 amplification, FGF3 amplification, FGF4 amplification, MYST3 amplification
16	Ductal	Positive	Positive	Negative	Breast	PTEN mutation E7fs*9, BRCA2 mutation K3326*, CCND1 amplification, EP300 mutation P925T, FGF19 amplification, FGF3 amplification, FGF4 amplification
17	Ductal	Positive	Positive	Negative	Breast	ERBB4 amplification, HGF amplification, RICTOR amplification BCL2L2 amplification, JUN amplification, FGF10 amplification
18	Ductal	Positive	Negative	Negative	Abdomen	FGFR3 amplification, PIK3CA mutation H1047L, CCND1 amplification, TP53 mutation R249M
19	Papillary	Negative	negative	Negative	Lymph node	TP53 mutation S313fs*34, CCND1 amplification, FGF19 amplification, FGF3 amplification, FGF4 amplification
20	Ductal	Positive	Negative	Negative	Liver	PIK3CA mutation E542K, CCND1 amplification, MCL1 amplification, FGF19 amplification, FGF3 amplification, FGF4 amplification, GATA3 mutation I362fs*49+, MAP2K4 mutation E299*
21	Ductal	Positive	Not Known	Negative	Breast	PIK3CA mutation E545K, PIK3CA mutation Q546H, ATM mutation R2832C, CCND1 amplification, ARID1A mutation R1528*, FGF19 amplification, FGF3 amplification, FGF4 amplification
22	Ductal	Positive	Negative	Negative	Breast	PTEN loss, KRAS amplification, MYC amplification, CCND2 amplification, TP53 mutation V272M, RB1 loss, FGF23 amplification, FGF6 amplification, KDM5A amplification
23	Ductal	Positive	Positive	Negative	Breast	PTEN splice 1087_1088ins47+, TSC2 mutation N363fs*29, FGFR1 amplification, CCND1 amplification, MDM2 amplification, MYC amplification, TP53 mutation P278T, MDM4 amplification, FGF19 amplification, FGF3 amplification, FGF4 amplification, MYST3 amplification, ZNF703 amplification
24	Ductal	Positive	Negative	Negative	Breast	CCND1 amplification, Ep300 mutation P925T, FGF19 amplification, FGF4 amplification

### FGFR/FGF amplification

A complete list of FGFR and FGF amplifications is listed in Table 2. Of 112 patients, 24 (21%) had at least one amplified FGFR or FGF. Twelve of the 24 patients (50%) had more than one amplification. The presence of FGFR/FGF amplification was not significantly associated with age, ethnicity, hormone receptor status, HER2 status or site of mutation analysis (primary vs. metastatic tumor tissue).

**Table 2:** FGF and FGFR amplifications identified in 112 patients with metastatic breast cancer

Gene	# of patients with amplification (% of all patients)
FGFR1	8 (7%)
FGFR2	3 (3%)
FGFR3	1 (<1%)
FGF3	10 (9%)
FGF4	10 (9%)
FGF6	2 (2%)
FGF10	1 (<1%)
FGF14	1 (<1%)
FGF19	10 (9%)
FGF23	3 (3%)

The most common amplification was in *FGFR1*, seen in 8 (7%) patients. Five of the eight (63%) patients were hormone-receptor (estrogen and/or progesterone) positive. Three (3%) patients had an amplification in *FGFR2*, all of whom were triple-negative. *FGFR3* amplification was seen one patient (>1%) who was estrogen-receptor positive. *FGFR4* amplification was not seen.

Amplification in *FGF3*, *FGF4*, and *FGF19* appeared simultaneously in 10 (9%) patients. Three patients had amplification in *FGF23*, two patients had amplification in *FGF6* and amplification in *FGF10*, and *FGF14* were seen in one patient each.

### Simultaneous alterations

Of the 24 patients with amplification in FGFR or FGF, 15 (63%) also had an amplification in *CCND1*. All 10 patients with amplification in *FGF3*, *FGF4* and *FGF19* also had amplification in *CCND1*. Fifteen patients (63%) with an FGF/FGFR also had an alteration affecting the PI3K/AKT/mTOR pathway, including alterations in *PIK3CA*, *AKT*, *PTEN* and *NF1*. There was not a statistical association between alterations in the PI3K/AKT/mTOR pathway and the presence of FGFR/FGF amplifications (*P*=0.49). None of the patients with amplification in FGFR or FGF had amplification in *HER2*.

### Response to targeted therapies

Three of the 112 patients in this study received a non-selective FGFR inhibitor, including two of the 24 patients (8%) with FGFR or FGF amplification. Both of these patients had amplification of *FGF3*, *FGF4* and *FGF19*. A response was not seen with this treatment. One patient with *FGFR3* amplification was treated with the tyrosine kinase inhibitor pazopanib, that has some FGFR activity, with no response seen. Eleven of the 15 patients with FGF/FGFR amplification and an alteration in the PI3K/AKT/mTOR pathway received therapy targeting the PI3K/AKT/mTOR pathway and were evaluable for a response. Eight of the eleven patients (73%) experienced stable disease (SD) ≥ 6 months/partial response (PR)/ complete response (CR). In comparison, of 35 patients without FGF/FGFR amplification who had an alteration in the PI3K/AKT/mTOR pathway were treated with a therapy targeting this pathway and were evaluable for a response, 12 (34%) experienced SD≥6 months/PR/CR (*P*=0.0376). The TTF for patients with both type of alterations was 6.8 months (95% CI 2.413-11.187) compared to 3.7 months ( 95% CI 2.39-5.01) patients with only alterations in the PI3K/AKT/mTOR pathway (*P*=0.053). Seven patients with FGF/FGFR amplification and no alterations in the PI3K/AKT/mTOR pathway received an inhibitor of this pathway and were evaluable for a response and one (14%) experienced SD≥6 months/PR/CR.

## DISCUSSION

In our study, we observed FGFR and FGF amplification in 21% of patients with metastatic breast cancer who underwent NGS profiling. No mutations or fusions in FGFR were seen, consistent with previous reports that these classes of alterations are rare in breast cancer. [7] FGFR and FGF amplification, however, were common in our patient population, with 21% of patients demonstrating amplification.

The most common amplification was in *FGFR1*, observed in 7% of patients. It has been previously reported that *FGFR1* amplifications occur predominately in HR-positive patients [9], however; we observed similar rates of *FGFR1* amplifications in HR-positive and HR-negative patients (9% of HR-positive patients had an *FGFR1* amplification and 5% of HR-negative patients had an *FGFR1* amplification). This may have been attributable in part to our small study size.

Our data is consistent with previous reports demonstrating the co-existence of amplifications in the 11q12-14 amplicon. This amplicon contains *FGF3*, *FGF4*, *FGF19*, and *CCND1*. The simultaneous amplifications in *FGF3*, *FGF4*, *FGF19*, and *CCND1* have been previously reported [10]. In our analysis 10 of 112 patients demonstrated amplification in *FGF3, FGF4, FGF19* and *CCND1*. An additional patient had these amplifications as well had an amplification in *FGFR1*. We observed simultaneous amplification in *FGFR1* and *CCND1* in five of eight patients with an *FGFR1* amplification [11].

We observed *FGFR2* amplification exclusively in patients with triple-negative breast cancer (3 patients), consistent with previous reports [2, 12]. *FGFR3* and *FGFR4* amplification are less common than *FGFR1* and *FGFR2* in breast cancer [3, 13]. Consistent with these reports, we observed one *FGFR3* amplification among all patients, in a patient who with HR-positive breast cancer. We did not observe *FGFR4* amplification.

**Figure 1:**
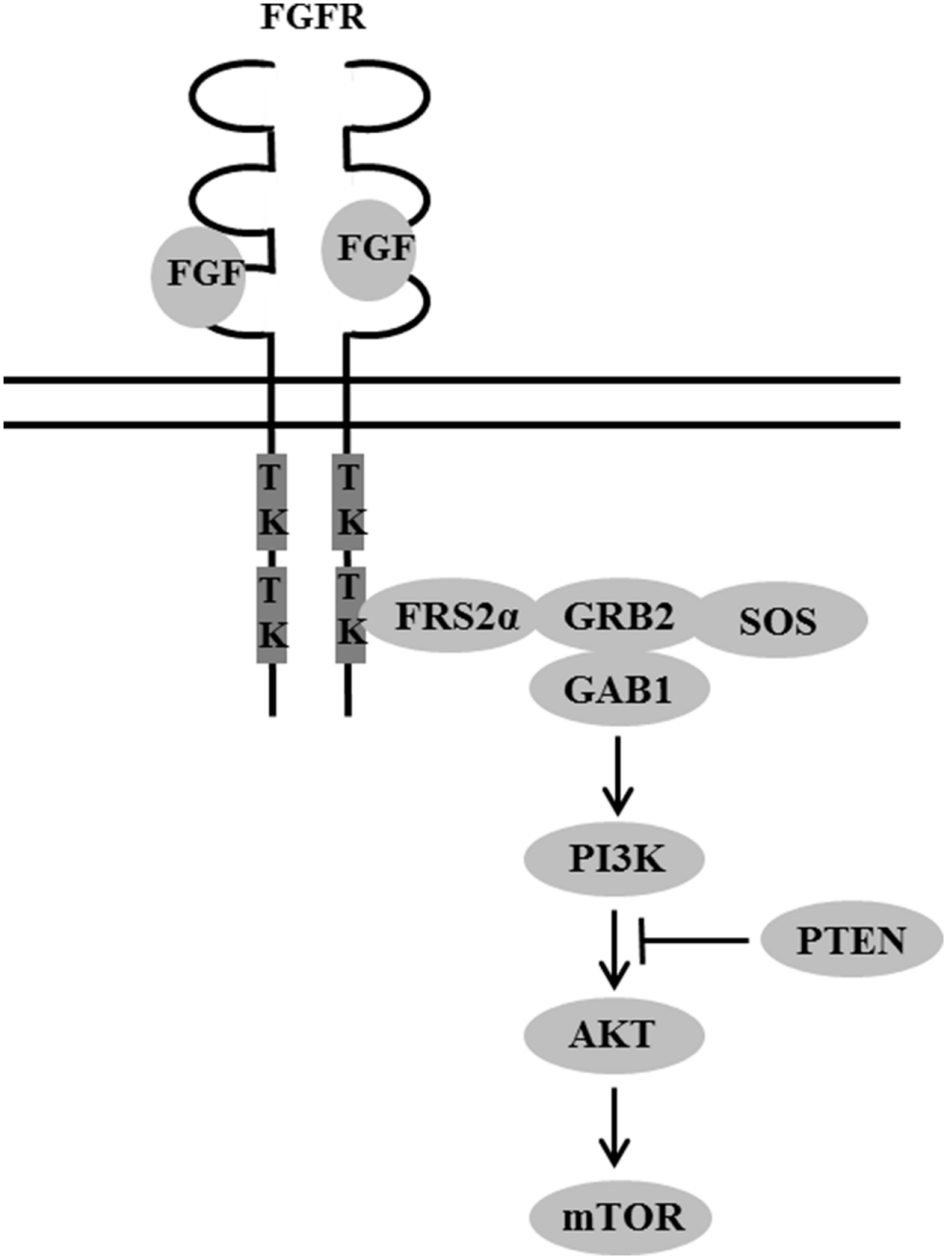
FGFR interaction with PI3K/AKT/mTOR pathway TK, tyrosine kinase domains; FRS2α, fibroblast growth factor receptor substrate 2α.

We observed that patients with simultaneous amplification in FGFR/FGF and alterations in the PI3K/ AKT/mTOR pathway had a higher rate of SD≥6 months/ PR/CR and TTF when treated with therapies targeting the PI3K/AKT/mTOR pathway than patients with alterations in the PI3K/AKT/mTOR pathway. This difference was statistically significant (73% vs. 34%; *P*=0.0376). Since FGFR/FGF signaling is known to activate the PI3K/AKT/ mTOR pathway (Figure 1) [14], tumors with simultaneous alterations may be more dependent on or “addicted” to this pathway for growth and survival, making it an attractive target for tumors with both of these types of alterations.

Previous studies have shown that activation of *AKT* is at least partially responsible for resistance to FGFR inhibitors in mammary and gastric cell lines with amplified FGFR levels [15, 16]. It was also determined that the combination of an FGFR inhibitor with rapamycin (a mTOR inhibitor) enhances the anti-proliferative effects in FGFR-addicted cells, suggesting that the combination of FGFR inhibitors with inhibitors of the PI3K/AKT/mTOR pathway may be an effective strategy for clinical development [15]. Notably, the two patients in our analysis who experienced clinical progression while receiving a nonselective FGFR inhibitor also had activating mutations in *PIK3CA*. We hypothesize that a lack of a clinical response in these patients could be due to an overactive PI3K/AKT/mTOR pathway. Only 14% of the patients (1 of 7) with an FGF/FGFR amplification, but no PI3K/AKT/mTOR alteration, who were treated with an inhibitor of the PI3K/AKT/mTOR pathway experienced SD≥6 months/PR/CR, which suggests therapy targeting this pathway may not be effective in tumors with in FGF/ FGFR alone.

This study has several limitations, including its small sample size. In addition, at the time these patients were treated there was limited availability of FGFR-directed therapies. Only four patients were treated with FGFR inhibitors. Another limitation is that this study represents retrospective data. Unfortunately, results of the hybrid capture based NGS were typically not used in selection of targeted therapies. A prospective study allowing for selection of matched therapies would more accurately reflect associations between these molecular alterations and response. Genomic profiling was performed on available tissue that was either from the primary tumor or a metastatic site. Ideally, both primary and metastatic tissue would be analyzed with the approach described herein. Pre- and post-treatment biopsies are also helpful to identify changes in molecular profile that occur over time and in response to therapy.

Despite the limitations of this study, our data suggests that concomitant presence of FGFR/FGF amplification and alterations in the setting of an activated PI3K/AKT/mTOR pathway may predict for better outcomes to PI3K/AKT/mTOR inhibitors. Given the prevalence of PI3K/AKT/mTOR pathway alterations in patients with breast cancer [17-19] and the availability of several inhibitors of this pathway, this coexistence of molecular alterations may be an important biomarker. It is also suggestive that combination approaches to treatment that include both an FGFR inhibitor and a PI3K/AKT/ mTOR inhibitor may be beneficial. Further studies in larger groups of patients are ongoing.

## PATIENTS AND METHODS

### Patients

Patients with advanced and metastatic breast cancers who experienced treatment failure with standard therapy and who had tissue available for mutation analysis were eligible. The study was conducted in the Department of Investigational Cancer Therapeutics (Phase I Clinical Trials Program) at MD Anderson Cancer Center. The registration of patients in the database and pathology assessment was performed at MD Anderson. Hybrid capture based, comprehensive next-generation sequencing of tumor tissue was performed at Foundation Medicine using FoundationOne™ (Cambridge, MA). Eligible patients were those referred for phase I clinical trials for targeted therapeutic agents. This study and all treatments were conducted in accordance with the guidelines of the MD Anderson Institutional Review Board. Some patients had their tumors analyzed prospectively and were enrolled on trials according to molecular profile, while others had their tumors analyzed retrospectively after already being enrolled on a trial.

### Evaluation of HER2 amplification, estrogen and progesterone receptor status, PTEN protein loss

Under CLIA conditions, immunohistochemistry was used to measure of HER2, estrogen and progesterone receptors and the presence of PTEN protein loss. Estrogen and progesterone receptors were assessed using antibody 6F11 (Novocastra Laboratories, Ltd., Newcastle Upon Tyne, UK). Alternatively, fluorescence in situ hybridization (FISH) was used to measure the copy number of HER2 according to current guidelines.

### Hybrid captured based comprehensive next-generation sequencing

Tumor samples were evaluated for genomic alterations including base substitutions, short insertions and deletions, amplifications homozygous deletions, gene fusions, truncations and rearrangements (Foundation Medicine, Cambridge, MA). DNA was extracted from 40 μm of FFPE tissue (minimum 20% tumor cells) using the Maxwell 16 FFPE Plus LEV DNA Purification kit (Promega) and quantified using a standardized PicoGreen fluorescence assay (Invitrogen). Library Construction was performed using 50-200 ng of DNA sheared by sonication to ∼100-400 bp before end-repair, dA addition and ligation of indexed, Illumina sequencing adaptors. Enrichment of target sequences (all coding exons of 182 or 236 cancer-related genes and selected introns from 14 or 19 genes recurrently rearranged in cancer) was achieved by solution-based hybrid capture with custom biotinylated oligonucleotide baits. Enriched libraries were sequenced to an average median depth of >500X with 99% of bases covered >100X (Illumina HiSeq 2000 platform using 49 × 49 paired-end reads) and mapped to the reference human genome (hg19) using the Burrows-Wheeler Aligner and the publicly available SAMtools, Picard and Genome Analysis Toolkit. Point mutations were identified by a Bayesian algorithm; short insertions and deletions, determined by local assembly; gene copy number alterations (amplifi by comparison to process matched normal controls; and gene fusions/ rearrangements, by clustering chimeric reads mapped to targeted introns. Local site permissions to use clinical samples were also obtained. Genes were considered amplified if the copy number was amplified 5 times for *ERBB2* (*HER2*) and 6 times for all other genes.

### Treatment and evaluation

Assignment to a clinical trial was determined after clinical, laboratory, and pathologic data from all available patient records were reviewed. Consecutive patients who had tumor tissue that could be tested or had been tested with underlying alterations were enrolled, whenever possible, in clinical trials that directly targeted that alteration. Some patients, whose tumors were analyzed for alterations retrospectively, were also enrolled in clinical trials without such knowledge. Treatment continued until disease progression or unacceptable toxicity occurred. Treatment was carried out according to the specific requisites in the treatment protocols selected.

Assessments, including history, physical examination, and laboratory evaluations, were performed as specified in each protocol, typically before the initiation of therapy, weekly during the first cycle, and then, at a minimum, at the beginning of each new treatment cycle. Efficacy was assessed using computed tomography scans and/or magnetic resonance imaging at baseline before treatment initiation and then every two cycles (6 to 8 weeks). All radiographs were read in the Department of Investigational Cancer Therapeutics tumor measurement clinic. Responses were categorized per Response Evaluation Criteria in Solid Tumors (RECIST) and were reported as best response [20]. In brief, complete response (CR) was defined as the disappearance of all measurable and nonmeasurable disease. Partial response (PR) was defined as at least a 30% decrease in the sum of the longest diameter of measurable target lesions. Progressive disease (PD) was defined as at least a 20% increase in the sum of the longest diameter of measurable target lesions, unequivocal progression of a nontarget lesion, or the appearance of a new lesion. Stable disease (SD) was defined as neither sufficient shrinkage to qualify for PR nor sufficient increase to qualify for PD. A confirmation of CR/PR required repeat imaging at least 28 days after the initial response assessment.

### Statistical analysis

Two-way contingency tables were formed to summarize the relationship between two categorical variables. The Fisher's exact test was used to assess the association among categorical variables and alteration status. Time to treatment failure (TTF) was defined as the time interval from the start of therapy to the termination of treatment for any reason, including withdrawal of patient consent, uncontrolled toxicities, disease progression or death. Patients still receiving treatment at the time of analysis were censored at the last follow-up date. Median TTF was estimated using the method of Kaplan and Meier and were compared among subgroups of patients using a log-rank test. All tests were two-sided, and *P*<0.05 was considered statistically significant. All statistical analyses were carried out using SPSS 17 software (SPSS, Chicago, IN, USA).
